# Probing Functional Changes in Exocyst Configuration with Monoclonal Antibodies

**DOI:** 10.3389/fcell.2016.00051

**Published:** 2016-06-03

**Authors:** Shivangi M. Inamdar, Shu-Chan Hsu, Charles Yeaman

**Affiliations:** ^1^Molecular and Cellular Biology Program, University of IowaIowa City, IA, USA; ^2^Department of Anatomy and Cell Biology, University of IowaIowa City, IA, USA; ^3^Department of Cell Biology and Neuroscience, Rutgers UniversityPiscataway, NJ, USA

**Keywords:** exocyst, monoclonal antibody, mammals, epithelium, cell polarity, membrane trafficking

## Abstract

Spatial regulation of exocytosis relies on the exocyst, a hetero-octameric protein complex that tethers vesicles to fusion sites at the plasma membrane. Nevertheless, our understanding of mechanisms regulating exocyst assembly/disassembly, localization, and function are incomplete. Here, we have exploited a panel of anti-Sec6 monoclonal antibodies (mAbs) to probe possible configurational changes accompanying transitions in exocyst function in epithelial MDCK cells. Sec6 is quantitatively associated with Sec8 in high molecular weight complexes, as shown by gel filtration and co-immunoprecipitation studies. We mapped epitopes recognized by more than 20 distinct mAbs to one of six Sec6 segments. Surprisingly, mAbs that bound epitopes in each segment labeled distinct subcellular structures. In general, antibodies to epitopes in N-terminal domains labeled Sec6 in either cytosolic or nuclear pools, whereas those that bound epitopes in C-terminal domains labeled membrane-associated Sec6. In this latter group, we identified antibodies that labeled distinct Sec6 populations at the apical junctional complex, desmosomes, endoplasmic reticulum and vimentin-type intermediate filaments. That each antibody was specific was verified by both Sec6 RNAi and competition with fusion proteins containing each domain. Comparison of non-polarized and polarized cells revealed that many Sec6 epitopes either redistribute or become concealed during epithelial polarization. Transitions in exocyst configurations may be regulated in part by the actions of Ral GTPases, because the exposure of Sec6 C-terminal domain epitopes at the plasma membrane is significantly reduced upon RalA RNAi. To determine whether spatio-temporal changes in epitope accessibility was correlated with differential stability of interactions between Sec6 and other exocyst subunits, we quantified relative amounts of each subunit that co-immunoprecipitated with Sec6 when antibodies to N-terminal or C-terminal epitopes were used. Antibodies to Sec6NT co-precipitated substantially more Sec5, -10, -15, Exo70 and -84 than did those to Sec6CT. In contrast, antibodies to Sec6CT co-precipitated more Sec3 and Sec8 than did those to Sec6NT. These results are consistent with a model in which exocyst activation during periods of rapid membrane expansion is accompanied by molecular rearrangements within the holocomplex or association with accessory proteins, which expose the Sec6 C-terminal domain when the complex is membrane-bound and conceal it when the complex is cytoplasmic.

## Introduction

Exocysts are multifunctional protein scaffolds that mediate vesicle tethering during exocytosis (Luo et al., 2014), but also function in many other membrane trafficking pathways during endocytic recycling and transcytosis (Jafar-Nejad et al., 2005; Oztan et al., 2007), cytokinesis (Fielding et al., 2005; Gromley et al., 2005; Cascone et al., 2008), ciliogenesis (Zuo et al., 2009; Das and Guo, 2011), and ciliary shedding (Overgaard et al., 2009), cell motility (Rosse et al., 2006; Zuo et al., 2006; Spiczka and Yeaman, 2008), autophagy (Bodemann et al., 2011; Simicek et al., 2013), membrane nanotube formation (Hase et al., 2009), invadopodia formation (Sakurai-Yageta et al., 2008; Liu et al., 2009), phagocytosis (Mohammadi and Isberg, 2013), and bacterial invasion into host cells (Nichols and Casanova, 2010). Not all activities that have been attributed to exocyst complexes involve the tethering of membranes, however. Additional functions for exocyst components have been described in tumor cell survival (Chien et al., 2006), innate immunity signaling (Ishikawa et al., 2009; Simicek et al., 2013), and DNA repair (Torres et al., 2015).

In order to coordinate exocyst activities in such a diversity of events, cells must exert precise spatio-temporal regulation over exocyst recruitment to different cellular compartments and association with additional factors that execute specific functions there. A growing list of exocyst-binding proteins is emerging (Sivaram et al., 2005; France et al., 2006; Medkova et al., 2006; Goehring et al., 2007; Jin et al., 2011; Parrini et al., 2011; Morgera et al., 2012; Dubuke et al., 2015), and for many of those that have been examined, their localization and function is dependent on recruitment to exocyst complexes at those sites (Fielding et al., 2005; Gromley et al., 2005). Most “accessory proteins” do not bind the exocyst consitutively, but do so in a manner that is regulated by either phosphorylation (Chen et al., 2011; Stalder and Novick, 2016) or, more commonly, small GTPases (Chien et al., 2006; Rittmeyer et al., 2008; Sakurai-Yageta et al., 2008; Spiczka and Yeaman, 2008; Lalli, 2009; Bodemann et al., 2011; Pathak et al., 2012; Das et al., 2014).

We hypothesize that exocysts are allosterically regulated scaffolds that expose or conceal binding sites for pathway-specific factors at different times and places within cells. By electron microscopy, purified endogenous exocyst complexes from *Saccharomyces cerevisiae* resemble a collection of long rods, which consist of tightly packed helical bundles that may represent individual subunits of the complex (Heider et al., 2016). This structure is consistent with images generated by quick-freeze/deep-etch electron microscopy of glutaraldehyde-fixed exocyst complexes purified from rat brain extracts (Hsu et al., 1996). However, in the same study non-fixed exocyst complexes appeared as flowers with four to six “petals” (Hsu et al., 1996). This has prompted speculation that exocyst complexes may exist in different conformational states (e.g., “open” vs. “closed”; Munson and Novick, 2006). If exocyst activities are regulated through either conformational changes within the holocomplex or spatio-temporal control of accessory protein binding to the holocomplex, then one prediction is that different epitopes on the complex would be exposed when one exocyst is at one site engaged in one activity, but concealed on another exocyst performing a different function at a different site.

Previous studies support this prediction. For example, distinct cohorts of monoclonal antibodies were reported to label different populations of exocyst complexes associated with plasma membrane and *trans*-Golgi network of normal rat kidney cells (Yeaman et al., 2001). In addition, polyclonal antibodies raised against either the N- or C-terminus of Sec3 labeled plasma membranes of epithelial MDCK cells, but only N-terminal specific antibodies labeled an additional population of Sec3 associated with a perinuclear compartment (Andersen and Yeaman, 2010). Depending on the specific antibody used, the degree of cellular polarization and the manner in which cells were permeabilized and fixed, Sec8 has been variously localized to different plasma membrane domains (Bryant et al., 2010), intercellular junctions (Grindstaff et al., 1998; Yeaman et al., 2004; Andersen and Yeaman, 2010), centrosomes (Rogers et al., 2004), and endosomal populations (Folsch et al., 2003; Prigent et al., 2003; Oztan et al., 2007) in MDCK cells. Finally, in a number of studies individual exocyst subunits have been reported to have distinct localization patterns (Vik-Mo et al., 2003; Beronja et al., 2005; Mehta et al., 2005; Bryant et al., 2010; Bodemann et al., 2011). Whether these results reflect functionally distinct exocyst sub-complexes or dynamic intermediates of holocomplex assembly and disassembly is not clear, and is difficult to reconcile with recent findings that exocyst exists predominantly as a stable hetero-octamer, at least in budding yeast (Heider et al., 2016).

Progress toward a thorough understanding of this essential complex and its diverse functions will require careful analysis of its myriad associations and how exocyst assembly, disassembly and activity is regulated. Although results of immunolocalization studies of this complex in mammalian cells must be interpreted cautiously, we hypothesize that careful analysis with a panel of monoclonal antibodies can reveal important details about exocyst functions and regulation during its involvement in a host of cellular activities.

## Material and methods

### Antibodies

Mouse monoclonal antibodies (mAbs) against Sec6 and Sec8 have either been described previously (Hsu et al., 1996; Kee et al., 1997; Yeaman et al., 2001) or were produced at the same time as previously described mAbs were. mAbs against Sec5, Exo70, and Exo84 were described previously (Vega and Hsu, 2001; Wang and Hsu, 2003; Spiczka and Yeaman, 2008), as was anti-CDC10 rabbit polyclonal antibody (Hsu et al., 1998). Rabbit polyclonal antibodies against Sec3, Sec5, Sec10, Sec15, Exo70, and Exo84 have also been described previously (Yeaman, 2003; Andersen and Yeaman, 2010). Rabbit polyclonal antibodies against Nop2 (NBP1-92192) and vimentin (NBP1-31327) were from Novus Biologica (Littleton, CO). Rabbit anti-desmoplakin-1/2 (ab14418) was from Abcam (Cambridge, MA), rabbit anti-pericentrin (PRB-432C) was from BioLegend (San Diego, CA), rabbit anti-β-tubulin (mAb 9F3) was from Cell Signaling Technology (Danvers, MA) and rabbit anti-calnexin (ADI-SPA-865) was from Enzo Life Sciences, Inc. (Farmingdale, NY). Fluorescein isothiocyanate (FITC)-goat anti-mouse, Texas Red (TR)-donkey anti-rabbit and Alexa594 goat anti-rabbit immunoglobulin (Ig)G were purchased from Jackson ImmunoResearch Laboratories (West Grove, PA). Horseradish peroxidase-conjugated goat anti-mouse and goat anti-rabbit antibodies were purchased from Promega (Madison, WI).

### Cell culture

Madin-Darby Canine Kidney (MDCK) strain II cells were maintained in low-glucose Dulbecco's modified Eagle's medium (LG-DMEM) with 1.8 mM Ca^2+^ containing 1 g/l sodium bicarbonate and supplemented with 10% fetal bovine serum (FBS; Cell Generation, Fort Collins, CO), penicillin, streptomycin, and gentamicin (PSG) and grown at 37°C with 5% CO_2_. For disruption of microtubules or actin, cells were incubated in medium containing either 20 μg/ml nocodazole (Sigma, St. Louis, MO) for 2 h (Reaves and Banting, 1992), or 2 μM cytochalasin D for 60 min (Stevenson and Begg, 1994).

shSec6 cells were generated by stable integration of short hairpin RNAs (shRNAs) targeting canine Sec6 (sense: 5′-GCTGCTCAGATAAGTGAAGAT-3′). shRNA was delivered via transduction with a recombinant lentiviral vector that was pseudotyped with vesicular stomatitis virus G protein. Cells were selected and maintained in medium containing 5 μg/ml puromycin. Negative controls were generated by transducing MDCK II cells with lentiviral vectors encoding a non-targeting shRNA (sense: 5′-CCAGACCTTCAAGGAATCCAT-3′) and selecting them in puromycin. Sec6 rescue cells were generated by introducing 3 wobble-base point mutations into the first three codons of the target Sec6 hairpin sequence of canine Sec6 cDNA. This cDNA was ligated into pQCXIN and delivered into shSec6 cells via retroviral transduction. Transfected cells were selected using 400 μg/ml G418 and assayed by immunofluorescence and immunoblotting for hairpin resistant (hr) Sec6. shCtrl, shRalA and shRalB MDCK II cells were described previously (Hazelett et al., 2011).

Three-dimensional cysts of MDCK cells were generated as described (Bryant et al., 2010). Prior to seeding cells, a thin layer of growth factor-reduced Matrigel (BD Biosciences, Bedford, MA) was spread into 8-well Lab-Tek Chambered Coverglass slides and placed at 37°C for 10 min. Cells were trypsinized and diluted in ice-cold media to a concentration of 4 × 10^4^ cells/ml. Matrigel was diluted in ice-cold media to 4%. Matrigel solutions and cell suspensions were mixed 1:1 yielding 2 × 10^4^ cells/ml in a 2% Matrigel solution. Solutions were then added to wells of chambered coverglass slides and placed at 37°C for 4 days.

### Epitope mapping

**Figure 1:**
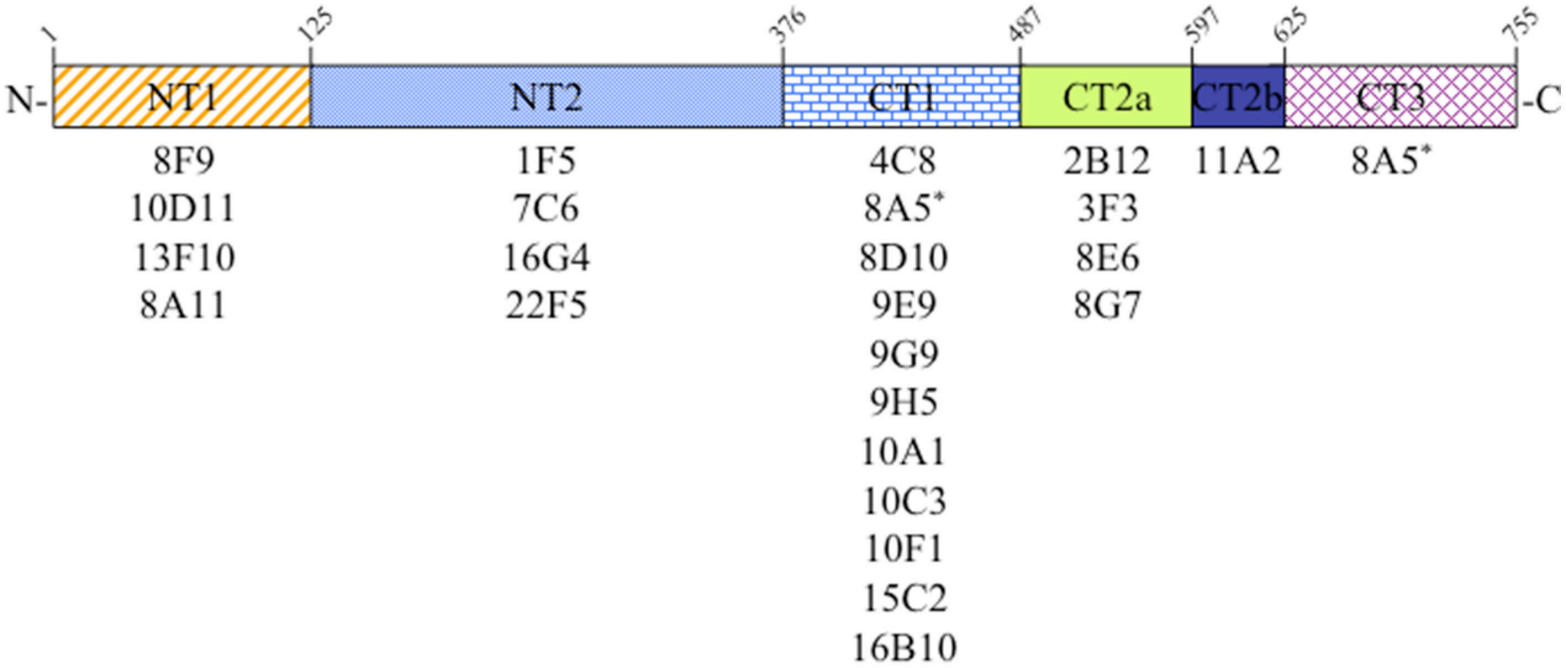
**Epitope mapping analysis of Sec6 monoclonal antibodies**. Antibodies produced by 24 different hybridomas were tested for binding specificity to seven recombinant Sec6 fragments, defining 6 distinct protein segments. Amino acid residues marking the boundaries of these segments in rat Sec6 are shown above each. See text for details.

Sequences from a cDNA encoding rat Sec6 were subcloned into the pGEX-KG vector to express as GST-fusion proteins in *E. coli*. Initially, four Sec6 fragments were generated, corresponding to amino acid residues 2–125 (Sec6.1 = NT1), 126–376 (Sec6.2 = NT2), 377–597 (Sec6.3), and 586–755 (Sec6.4). Subsequently, fragments of the C-terminal half were redesigned based on structural characterization of yeast Sec6 (Songer and Munson, 2009) and expressed as CT1 (residues 377–487), CT2 (residues 488–625), and CT3 (residues 626–755). GST-fusion proteins were purified from bacterial lysates and serial three-fold dilutions were applied to Immobilon PVDF membrane (Millipore) and blocked with Blotto (5% nonfat dry milk, 0.5% normal goat serum, and 0.1% sodium azide in TBS) overnight at 4°C. Dot blots were incubated with primary antibodies overnight at 4°C, and then washed 5 times with TST (TBS containing 0.1% Tween-20), 10 min each. Finally, blots were incubated with HRP-labeled goat anti-mouse IgG for 45 min at room temperature and then washed as above. After 2 final washes with TBS, blots were incubated with SuperSignal West Pico Chemiluminescent Substrate (Thermo Scientific) and visualized with a ChemiDoc-It™ Imaging System and quantified with Visionworks Software (UVP; Upland, CA). Segments labeled CT2a and CT2b in Figure 1 were defined by comparing results obtained with the original Sec6.3/6.4 fragments and the redesigned Sec6 CT1/CT2/CT3 fragments. Because mAbs 2B12, 3F3, 8E6, and 8G7 bound both the Sec6.3 and CT2 fragments, whilst mAb 11A2 bound the Sec6.4 and CT2 fragments, it was deduced that these antibody groups bound different epitopes within the CT2 domain, herein labeled CT2a and CT2b.

### Immunofluorescent labeling

Samples were fixed on ice with either 100% methanol (MeOH) or 4% paraformaldehyde (PFA) for 20 min, either prior to or following extraction with CSK buffer (1% Triton X-100, 10 mM Pipes, pH 6.8, 50 mM NaCl, 300 mM sucrose, 3 mM MgCl_2_) supplemented with protease inhibitors (1 mM pefabloc and 10 μg/ml each of aprotinin, antipain, leupeptin, and pepstatin A) for 10 min. PFA-fixed samples were then quenched with Ringer's saline (154 mM NaCl, 1.8 mM Ca^2+^, 7.2 mM KCl, and 10 mM HEPES, pH 7.4) containing 50 mM NH_4_Cl. After samples were blocked with 0.2% fish skin gelatin in Ringer's saline (blocking buffer) for 1 h, primary antibodies were diluted in blocking buffer and applied for 1 h at room temperature or overnight at 4°C with gentle rocking. After 5 washes with blocking buffer, secondary antibodies and DAPI were applied for 30 min at room temperature. Samples were washed 5 times with blocking buffer, and filters and coverslips were mounted onto slides using Elvanol-PPD. For processing MDCK cysts, all buffers were at room temperature and secondary antibodies were applied for 1 h. Images were obtained using either a Zeiss 510 scanning confocal microscope (Thornwood, NY; 63X objective) equipped with a krypton/argon laser (FITC excitation using 488 nm laser line and Texas Red excitation using 543 laser line), or with a Leica DMI 6000B microscope equipped with a *BD CARV II* spinning disk confocal imager. Stacks of confocal images were collected from several different fields using a 63X objective and a Photometrics QuantEM 512SC EM-CCD high-speed camera.

For studies examining immunofluorescence intensity at the plasma membrane, serial optical sections were merged and antibody labeling intensities at lateral plasma membranes were quantified by tracing the outlines of ~50 cells from 5 representative fields and determining mean pixel intensities using the wand tool with ImageJ software. For each sample, values were normalized by dividing the pixel intensity of Sec6 labeling by that of Sec3 labeling. One-way ANOVA with Tukey's post-test statistical analyses were performed for each cell type, and differences were considered significant where *p* < 0.05.

### Immunoprecipitation

MDCK cells were washed 3 times with Ringer's saline on ice, and lysed in RIPA buffer (50 mM Tris-HCl pH 7.5, 1 % NP-40, 0.5% sodium deoxycholate, 150 mM NaCl, and 1 mM EDTA) containing protease inhibitors. Cell lysates were collected in 1.5 ml Eppendorf tubes and incubated on ice for 20 min at 4°C. Extracts were precleared with 5 μl of nonimmune serum and 50 μl *Staphylococcus aureus* cells (Pansorbin; Calbiochem Novabiochem, La Jolla, CA) for 1 h at 4°C. For Sec8 immunoprecipitation, mAbs 2E12, 5C3 and 10C2 were covalently cross-linked to Protein A Sepharose beads (Pharmacia LKB Nuclear, Gaithersburg, MD) with dimethyl pimelimidate (DMP), and 20 μl of immunoadsorbant was used per immunoprecipitation. For Sec6 NT or CT immunoprecipitations, combinations of either mABs that bind epitopes in NT1 or NT2 domains or those that bind epitopes in CT1, CT2, or CT3 domains were bound to Protein G Sepharose. Immunoadsorbants were incubated with pre-cleared cell extracts for 2 h at 4°C, then washed under stringent conditions and prepared for SDS-PAGE as described previously (Andersen and Yeaman, 2010).

### Superose 6 FPLC analysis

Confluent monolayers of MDCK cells were extracted for 10 min at 4°C in Tris-saline buffer containing 0.5% (v/v) NP-40 and protease inhibitors. Cell lysates were centrifuged at 15,000 × **g** for 10 min. The supernatant fraction was centrifuged at 100,000 × **g** for 30 min and passed through a 0.22 μm syringe filter (Millipore). Two hundred microliters of this lysate was applied to a Superose 6 HR 10/30 column and fractionated as described previously (Stewart and Nelson, 1997). Fractions 6–28 were separated by SDS-PAGE and proteins were electrophoretically transferred to Immobilon P membranes for immunoblotting with specific antibodies.

### Gel electrophoresis and immunoblotting

Protein samples were incubated in SDS-PAGE sample buffer for 10 min at 65°C before separation on 10% SDS polyacrylamide gels. Proteins were electrophoretically transferred onto Immobilon PVDF membrane (Millipore) and blocked with Blotto (5% nonfat dry milk, 0.5% normal goat serum, and 0.1% sodium azide in TBS) overnight at 4°C. Immunoblots were incubated with primary antibodies overnight at 4°C, and then washed 5 times with TST (TBS containing 0.1% Tween-20), 10 min each. Finally, immunoblots were incubated with HRP-labeled goat anti-mouse or goat anti-rabbit antibodies for 45 min at room temperature. Immunoblots were washed as above, then twice with TBS. Immunoblots were incubated with SuperSignal West Pico Chemiluminescent Substrate (Thermo Scientific) and visualized with a ChemiDoc-It™ Imaging System and quantified with Visionworks Software (UVP; Upland, CA).

## Results

### Epitope mapping defines distribution of antibody binding sites on Sec6

In the course of characterizing the mammalian exocyst complex, more than 30 monoclonal antibodies were generated against a His-tagged rSec6 fusion protein that specifically immunoblotted a single 86 kDa protein in rat brain homogenate (Hsu et al., 1996; Kee et al., 1997) and cell lines (Grindstaff et al., 1998; Yeaman et al., 2001). To conduct an informed analysis of Sec6 immunolocalization in epithelial cells, we first mapped epitopes recognized by 24 of these mAbs to one of seven recombinant Sec6 fragments, which defined six unique segments of the protein. Epitopes were distributed along the length of Sec6, and the nearly all of antibodies (23) bound a single segment (Figure 1). Most segments were detected by at least four mAbs, and the CT1 domain bound 11 mAbs. Intriguingly, one mAb (8A5) bound epitopes in both the CT1 and CT3 domains. That this hybridoma was monoclonal was confirmed by limiting dilution re-cloning of cells. We conclude that the epitope bound by mAb 8A5 is repeated in two segments of the Sec6 C-terminal domain.

### Antibodies to different Sec6 sements label distinct subcellular structures

**Figure 2:**
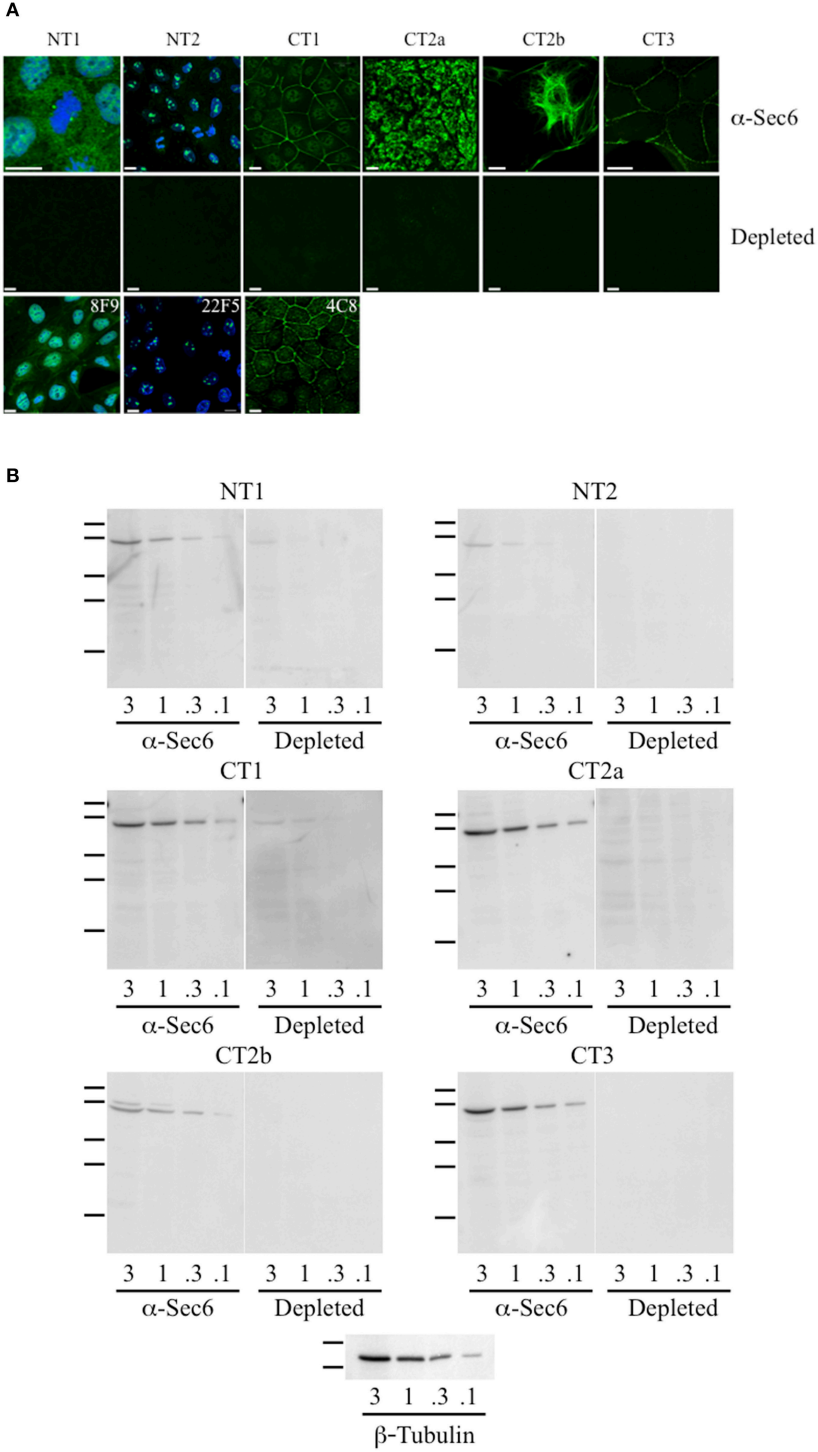
**Antibodies to different Sec6 subdomains label distinct subcellular structures. (A)** Immunofluorescent labeling of MDCK cells with antibodies to Sec6 NT1 (mAb 13F10), NT2 (mAb 16G4), CT1 (mAb 10C3), CT2a (mAb 3F3), CT2b (mAb 11A2), or CT3 (mAb 8A5) subdomains (“α-Sec6”) or hybridoma supernatants that were pre-incubated with recombinant Sec6 fragments containing epitopes recognized by those antibodies (“depleted”). Additional mAbs to Sec6 NT1 (mAb 8F9), NT2 (mAb 22F5), and CT1 (mAb 4C8) subdomains produced immunolocalization patterns that were indistinguishable from those of other mAbs that bound within the same subdomain. Cells were fixed with either 4% paraformaldehyde (NT1 and CT1) or 100% methanol (NT2, CT2a, CT2b, and CT3). For NT1 and NT2 labeling, cells were extracted with 1% Triton X-100 prior to fixation, and in top and bottom panels DNA was labeled with DAPI (blue). Bars = 10 μm. **(B)** Immunoblotting of Sec6 in detergent lysates of MDCK cells. Proteins (3, 1, 0.3, or 0.1 μg) were separated by SDS-PAGE and electrophoretically transferred to Immobilon P membranes. Membranes were probed with pooled antibodies to indicated Sec6 subdomains (“α-Sec6”), or with hybridoma supernatants that were pre-incubated with recombinant Sec6 fragments containing epitopes recognized by those antibodies (“Depleted”). An immunoblot probed for β-tubulin is shown as a loading control. Protein standards indicated are β-galactosidase (116 kDa), phosphorylase b (97 kDa), bovine serum albumin (66 kDa), egg albumin (45 kDa), and carbonic anhydrase (29 kDa).

**Figure 3:**
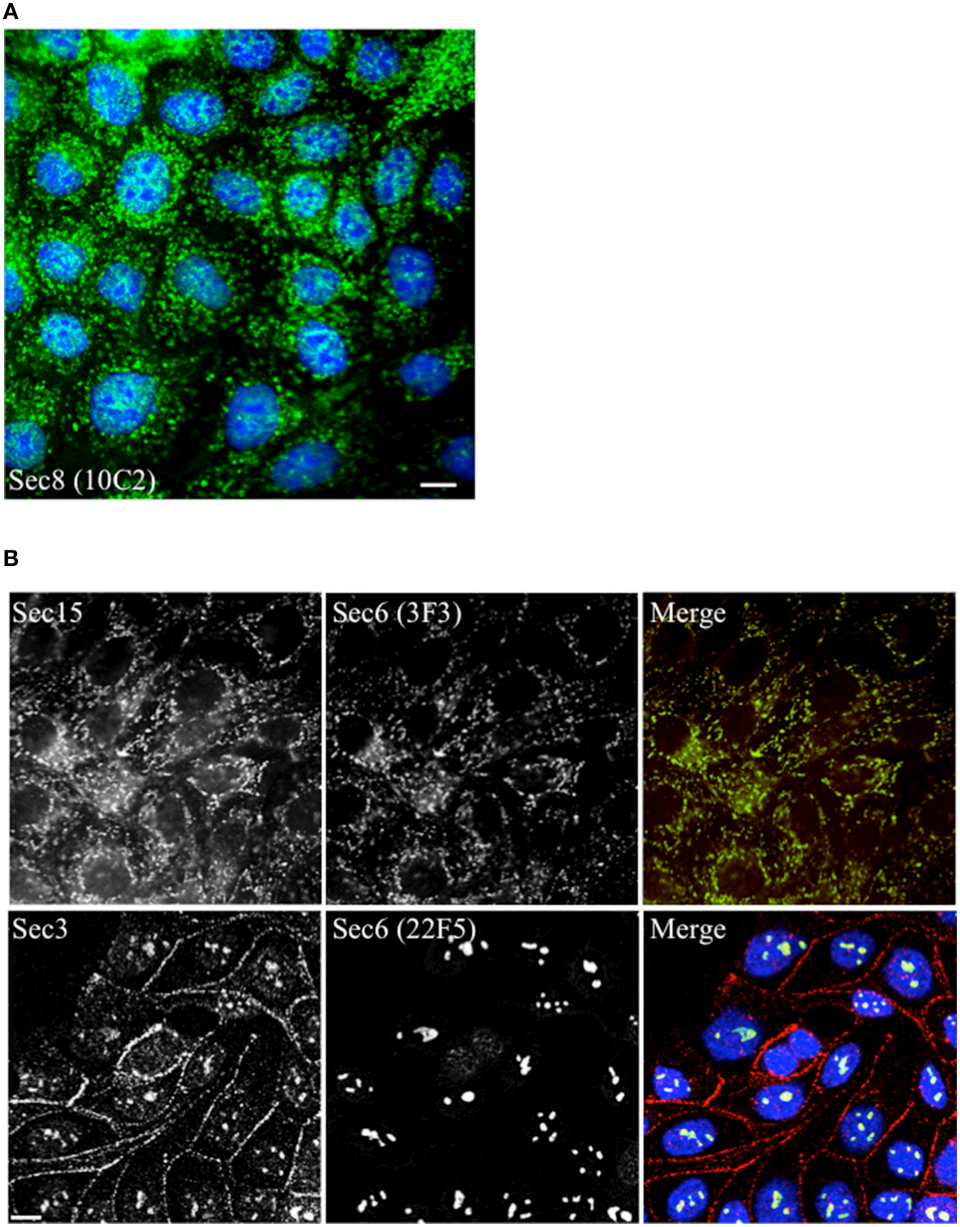
**Other exocyst subunits co-localize with Sec6 at sites labeled with subdomain-specific mAbs**. Immunofluorescent labeling of MDCK cells with mouse monoclonal antibodies to Sec8 [mAb 10C2, **(A)**, *green*] or Sec6 [either mAb 3F3, **(B)** top row, *green* or mAb 22F5, **(B)** bottom row, *green*] and rabbit polyclonal antibodies to Sec15 [Rb. 96, **(B)** top row, *red*] or Sec3 [Rb. 92, **(B)** bottom row, *red*]. Cells were fixed with either 100% methanol [**(A,B)**, top row] or were extracted with 1% Triton X-100 prior to fixation with 4% paraformaldehyde [**(B)**, bottom row]. DNA was labeled with DAPI (blue). Bars = 10 μm.

**Figure 4:**
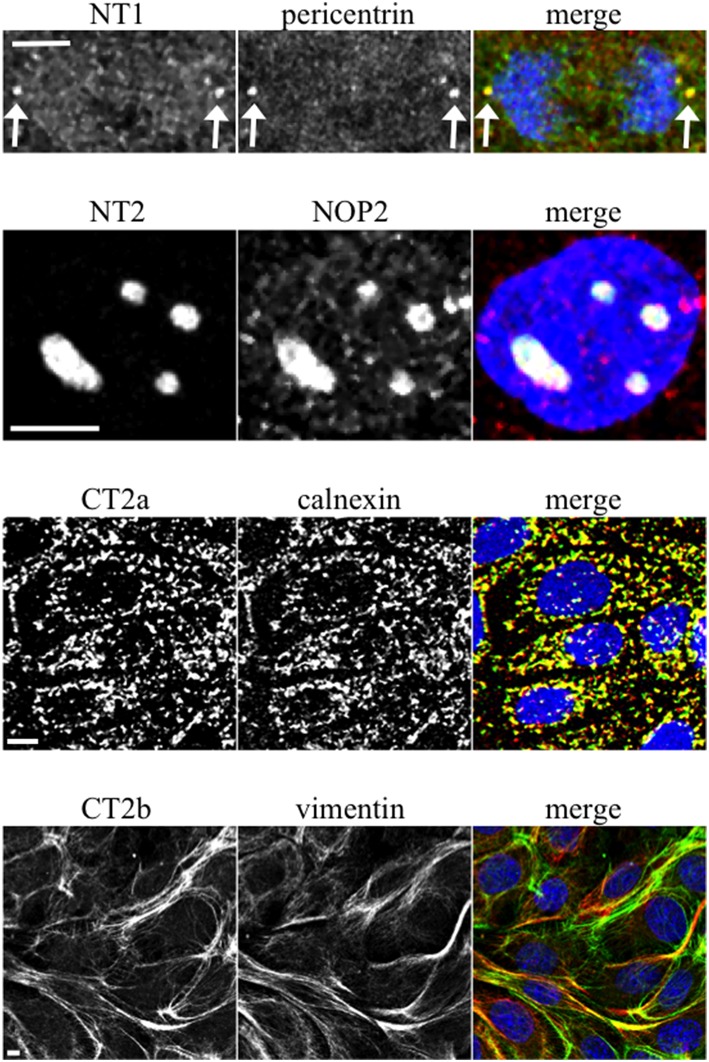
**Characterization of novel Sec6 localizations**. MDCK cells were fixed as described for Figure 2A and co-labeled with antibodies to Sec6 NT1 (mAb 13F10), NT2 (mAb 16G4), CT2a (mAb 3F3), or CT2b (mAb 11A2) (green) and antibodies to indicated proteins (red). In panels showing merged images, DNA was labeled with DAPI (blue). Bars = 5 μm.

When mAbs to different Sec6 segments were used to immunolabel epithelial cells, many different subcellular structures were revealed. Antibodies to the NT1 domain labeled plasma membrane weakly, but strongly labeled Sec6 in the cytosol and nucleus (Figure 2A). Sec8 mAb 10C2 labeled this subunit in the nucleus in a distribution similar to that seen with Sec6 NT1 antibodies (Figure 3). Sec6 NT1 epitopes were also detected on centrosomes, similar to localizations previously described for other exocyst subunits (Rogers et al., 2004; Zuo et al., 2009; Andersen and Yeaman, 2010). However, Sec6 NT1 mAbs only bound centrosomes in cells undergoing mitosis (Figures 2A, 4). Antibodies to epitopes in the NT2 domain also labeled nuclei, but in contrast to NT1 epitopes, those in the NT2 domain were concentrated in nucleoli (Figures 2A, 4). A population of Sec3 co-localized with this nucleolar-associated Sec6 pool (Figure 3). NT2-specific labeling became dispersed as nucleoli dissassembled, and labeling was not detected in mitotic cells (Figure 2A).

**Figure 5:**
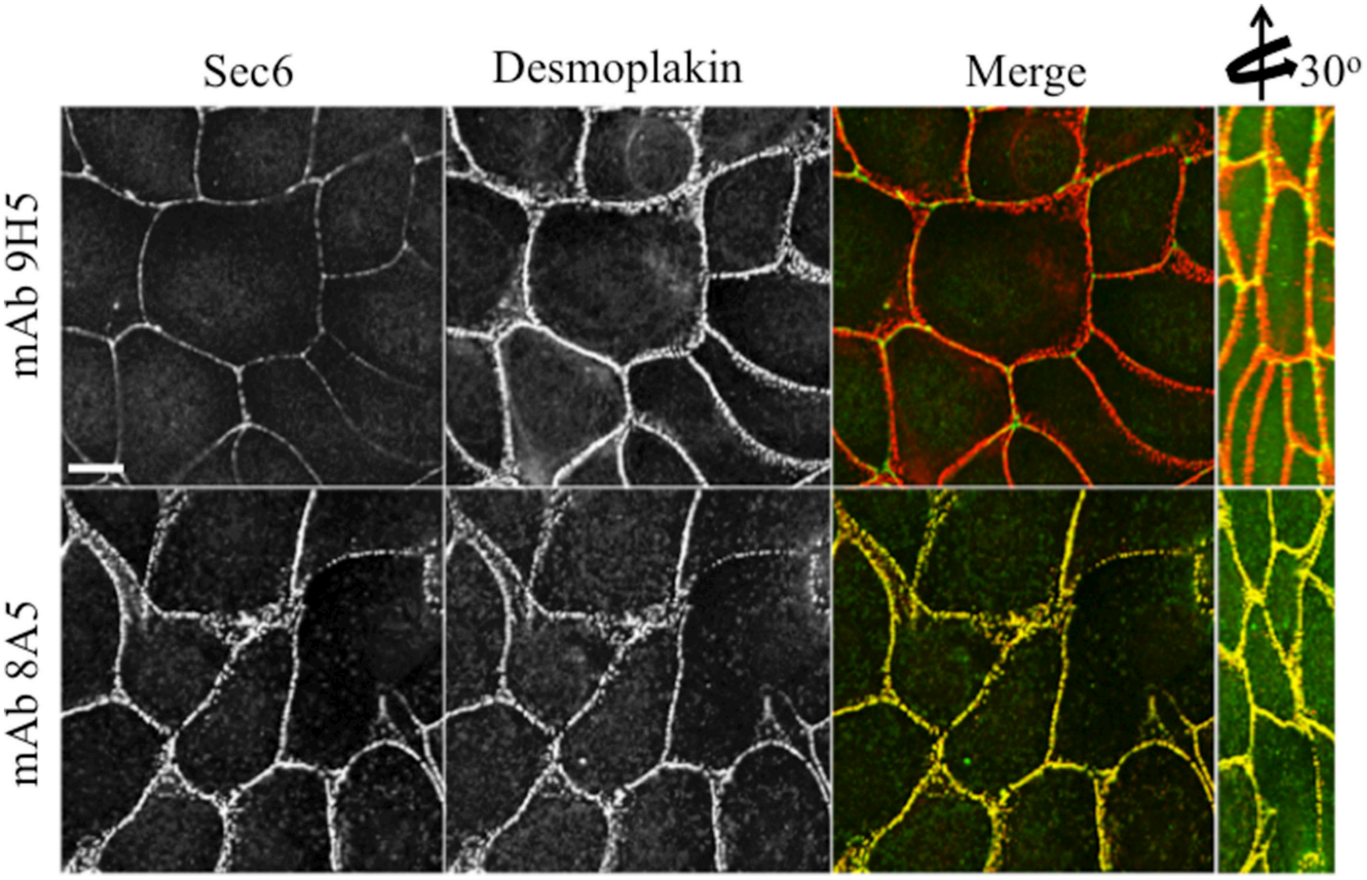
**Immunologically distinct pools of Sec6 are localized at AJC and desmosomes**. MDCK cells were fixed and labeled with indicated antibodies to Sec6 (green) and the desmosome component, desmoplakin (red). Note that mAb 8A5 detects a pool of Sec6 that co-localizes with desmoplakin. In contrast, mAb 9H5 detects a pool of Sec6 that does not co-localize with desmosome proteins. Bar = 10 μm.

In contrast to N-terminal specific antibodies, mAbs that bound epitopes within the C-terminal half of Sec6 mainly labeled membrane-associated complexes. Antibodies to Sec6 CT1 include mAb 9H5, which has previously been shown to label exocyst complexes associated with the apical junctional complex (AJC), which includes tight junctions and adherens junctions (Grindstaff et al., 1998; Yeaman et al., 2004; Figure 2A). This pool of Sec6 has previously been shown to co-localize with Sec8 (Grindstaff et al., 1998; Yeaman et al., 2004) and Sec10 (Lipschutz et al., 2000). Identical labeling patterns were observed with all of the Sec6 mAbs binding epitopes in this fragment, with the single exception of mAb 8A5. This antibody, which binds an epitope repeated in the CT3 domain, labels desmosomes but not other plasma membrane regions in non-polarized cells and polarized cells grown on Transwell filters (Andersen and Yeaman, 2010; Figures 2A, 5). As we reported previously, most exocyst subunits can be found at desmosomes (Andersen and Yeaman, 2010; also note membrane-associated pool of Sec3 in Figure 3).

**Figure 6:**
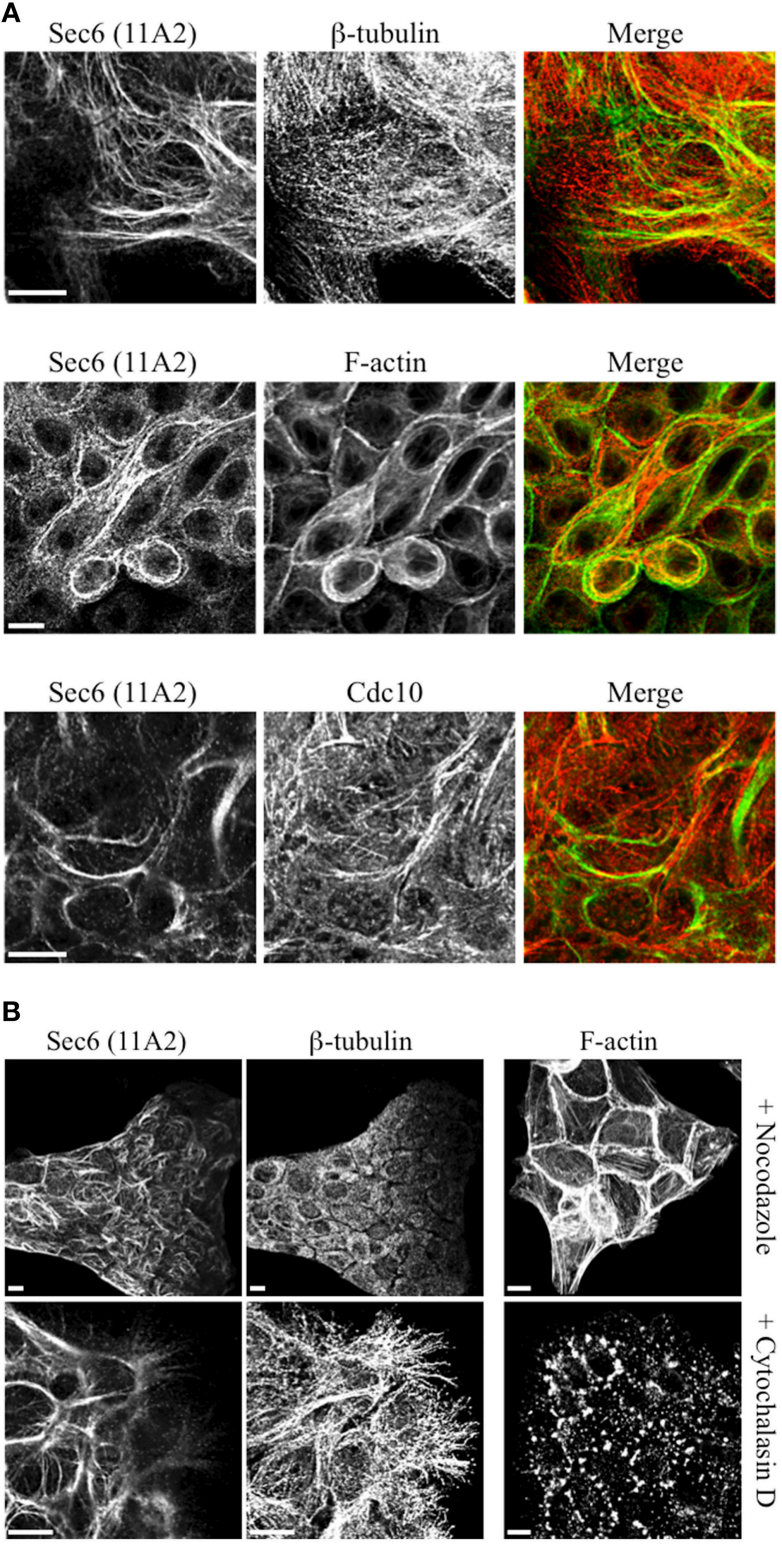
**Characterication of filamentous Sec6 mAb 11A2 labeling. (A)** MDCK cells were fixed with either 100% methanol (top and bottom rows) or 4% paraformaldehyde (middle row) and co-labeled with antibodies to Sec6 CT2b (mAb 11A2) and either β-tubulin or Cdc10 or with phalloidin. Bars = 10 μm. **(B)** MDCK cells were treated with either nocodazole or cytochalasin D, as described in Material and Methods. Cells were fixed with 100% methanol and co-labeled with antibodies to Sec6 CT2b (mAb 11A2) and β-tubulin. Panels on the right show cells that were fixed with 4% paraformaldehyde and labeled with phalloidin to demonstrate appearance of the actin cytoskeleton following treatment with drugs. Bars = 10 μm.

Several of the mAbs that bound epitopes within the CT2a domain did not label cells that had been fixed and permeabilized under any conditions tested, but one of them (mAb 3F3) strongly labeled internal membranes that were identified as endoplasmic reticulum (ER) by co-labeling with a calnexin-specific antibody (Figures 2A, 4). A similar labeling pattern was observed with select antibodies against both Sec8 and Sec15, supporting the conclusion that mAb 3F3 binds a population of Sec6 in ER-associated exocyst complexes (Figure 3). Finally, mAb 11A2, which binds a distinct epitope in the CT2 segment, revealed a pool of Sec6 that was associated with a filamentous cytoplasmic network (Figure 2A). This network was more extensively labeled with antibodies to vimentin (Figure 4) than it was with probes to other cytoskeletal networks, including microtubules, actin filaments, Cdc10-positive septin filaments (Figure 6A) and cytokeratins (not shown). Furthermore, although some overlap was observed between 11A2 labeling and microtubules and actin, filamentous Sec6 labeling was largely refractory to pharmacological disruption of those cytoskeletal structures with nocodazole or cytochalasin D, respectively (Figure 6B).

**Figure 7:**
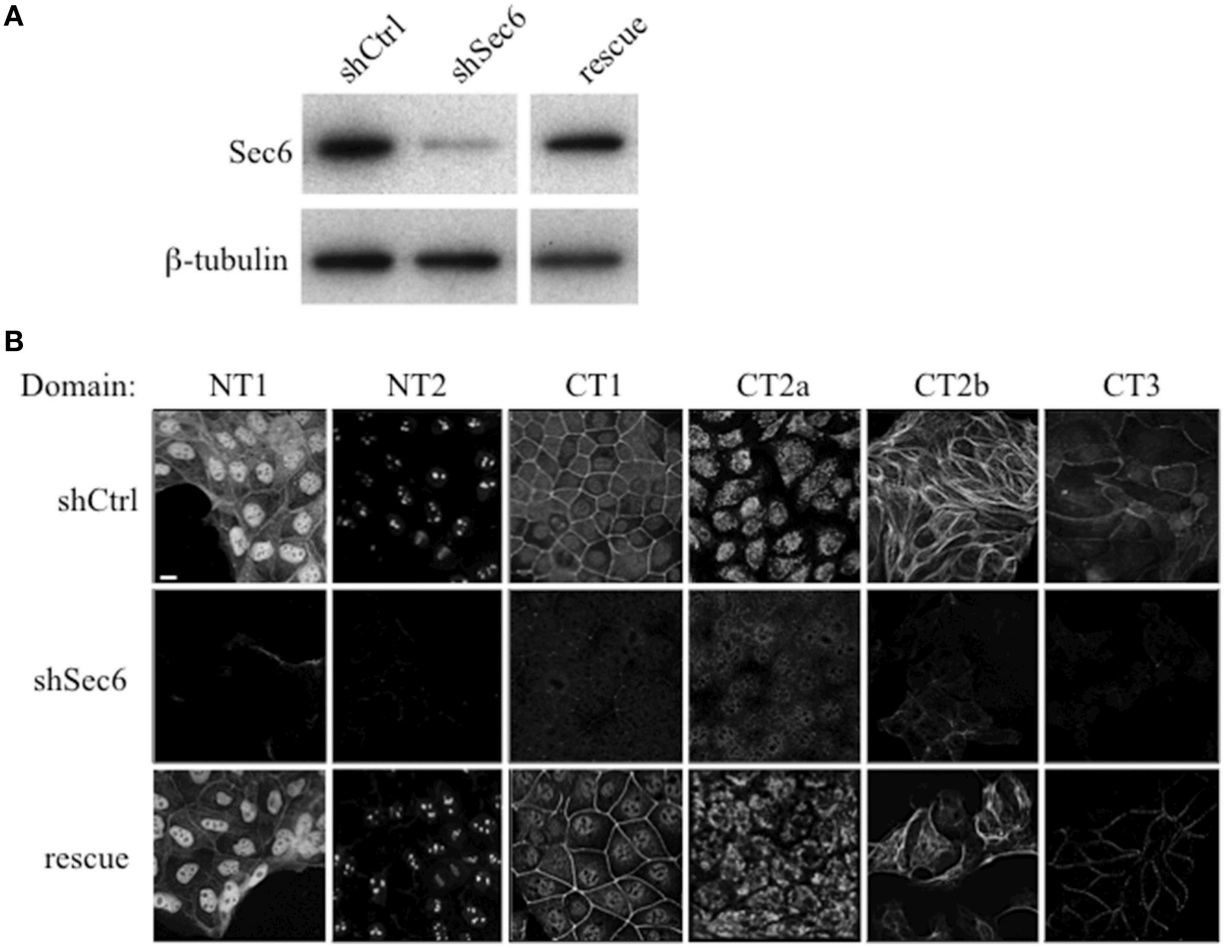
**Sec6 knockdown eliminates, and re-expression restores labeling with all Sec6 mAbs in MDCK cells. (A)** Sec6 knockdown and rescue. MDCK cells were transduced with recombinant lentiviral vectors carrying either non-targeting shRNA (shCtrl) or shRNA targeting Sec6 (shSec6). Immunoblot analysis confirmed that Sec6 protein expression was reduced in shSec6 cells by > 90% when normalized to loading control (β-tubulin). Rescue cell lines were established by transduction of shSec6 cells with recombinant retroviral vectors carrying hairpin-resistant Sec6 cDNA. Immunoblot analysis confirmed that Sec6 protein expression was restored to nearly identical levels as those in shCtrl cells. **(B)** Control (shCtrl), Sec6 knockdown (shSec6), and Sec6 rescue MDCK cells were processed for immunofluorescent labeling with antibodies to indicated Sec6 subdomains, as described in Figure 2. Bar = 10 μm.

To confirm that labeling with Sec6 mAbs was specific, two strategies were employed. First, immunodepletion of hybridoma supernatants with corresponding Sec6 fragments eliminated specific immunodetection of Sec6 on immunoblots (Figure 2B) and greatly reduced immunofluorescent staining associated with different subcellular compartments (Figure 2A). This confirmed that epitopes present in recombinant Sec6 fragments bound antibodies that were responsible for immunolabeling different organelles in cells. Cross-depletion of hybridoma supernatants with Sec6 fragments that did not contain associated epitopes did not diminish immunolabeling of cells (not shown). Second, RNAi-mediated reduction of Sec6 expression by >90% as verified by immunoblotting (Figure 7A) severely reduced or completely eliminated all labeling patterns observed (Figure 7B). This strongly supports the conclusion that Sec6 is the sole protein detected by all of the mAbs in cells. Finally, expression of a hairpin-resistant Sec6 cDNA (Figure 7A) restored immunolabeling of all of the observed compartments (Figure 7B), confirming not only that Sec6 is the protein recognized by all of the mAbs on diverse structures, but also that this diversity in labeling is not caused by alternatively spliced Sec6 variants localizing to distinct subcellular sites.

### Sec6 epitopes are differentially exposed or concealed during epithelial polarization

**Figure 8:**
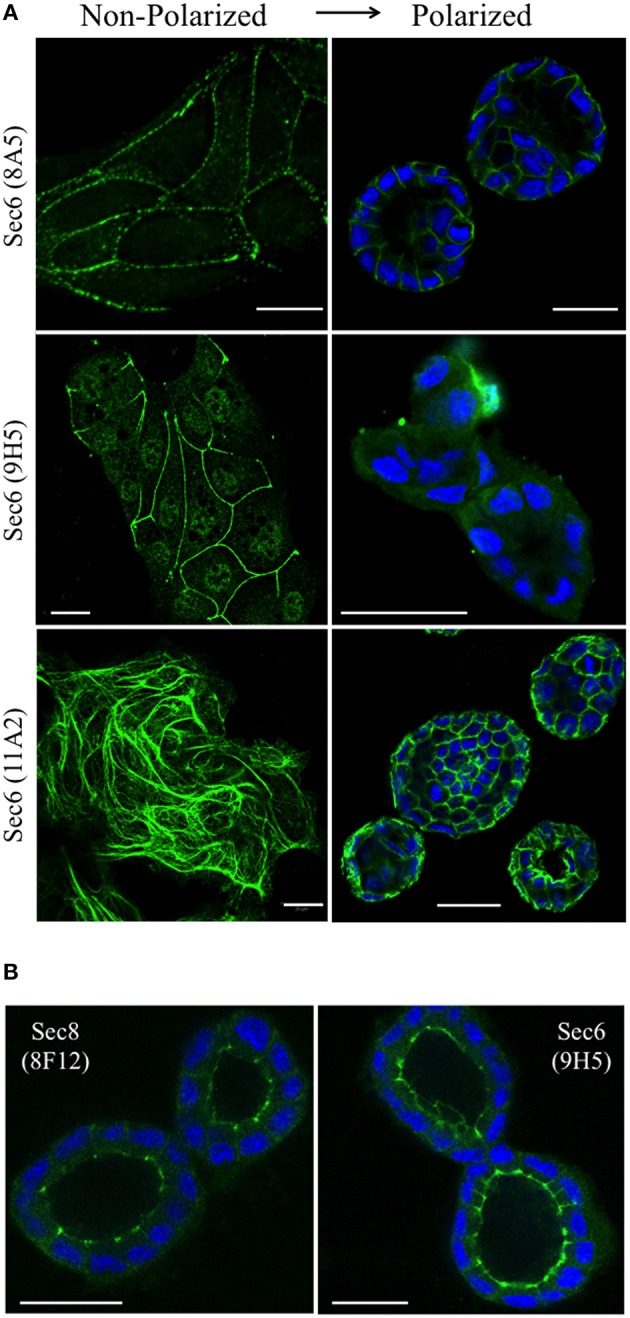
**Sec6 epitopes redistribute during epithelial polarization. (A)** MDCK cells were cultured as sub-confluent colonies on collagen-coated glass coverslips (non-polarized) or seeded in Matrigel and grown for 4 days (polarized). Cultures were fixed in 100% methanol and labeled with indicated anti-Sec6 mAbs (green). Polarized cultures were co-labeled with DAPI (blue). Bar = 20 μm. **(B)** MDCK cells were seeded in Matrigel and grown for 4 days. Cultures were either fixed in 100% methanol (left) or pre-extracted with 1% Triton X-100 before methanol fixation (right), and labeled with indicated antibodies (green) and DAPI (blue). Bar = 20 μm.

We next asked whether Sec6 epitopes were static, or whether they became exposed or concealed at different subcellular sites as cells underwent dynamic changes, as during morphogenesis of a three-dimensional cyst. We therefore compared localization profiles of epitopes bound by a subset of Sec6 mAbs in non-polarized and 3-D cysts of MDCK cells. When antibodies to epitopes in the C-terminal half of Sec6 were used, 2 patterns were observed. First, epitopes in the CT2b and CT3 segments appeared to redistribute during polarity development. In non-polarized and Transwell-cultured (2-D) polarized cells, mAb 8A5 labeled Sec6 in a discontinuous, punctate pattern characteristic of desmosomes, while mAb 11A2 labeled Sec6 on cytoskeletal filaments (Andersen and Yeaman, 2010; Figure 8A). In contrast, both antibodies revealed Sec6 in a uniform distribution along the basolateral plasma membrane of fully polarized cells in 3-D Matrigel cultures. In addition, mAb 11A2 detected Sec6 beneath the lumenal apical plasma membrane (Figure 8A). We note that a population of Sec6 remains associated with cytoplasmic filaments in 3-D cysts, but this pool is observed only when maximum intensity projections of stacked optical sections through the entire cyst are generated (not shown).

A second pattern was observed with antibodies to Sec6 CT1. Epitopes within this protein segment became concealed as cells developed polarity. For example, immunolabeling with mAb 9H5 was readily detected along lateral membranes of non-polarized cells (Figure 8A) and this became restricted to the AJC as cells acquired 2-D polarity when grown on Transwell filters (Grindstaff et al., 1998; Yeaman et al., 2004). However, specific Sec6 labeling was undetectable in fully polarized cells grown in Matrigel (Figure 8A). Importantly, Sec6 is still present at AJC in fully polarized cells, because its binding partner Sec8 was readily detected there and an AJC-associated pool of Sec6 was revealed when cells were gently extracted with non-ionic detergent prior to fixation (Figure 8B). However, the CT1 domain becomes masked at this site as cells undergo cystogenesis.

### Sec6 epitope accessibility is impacted by RalA activity

**Figure 9:**
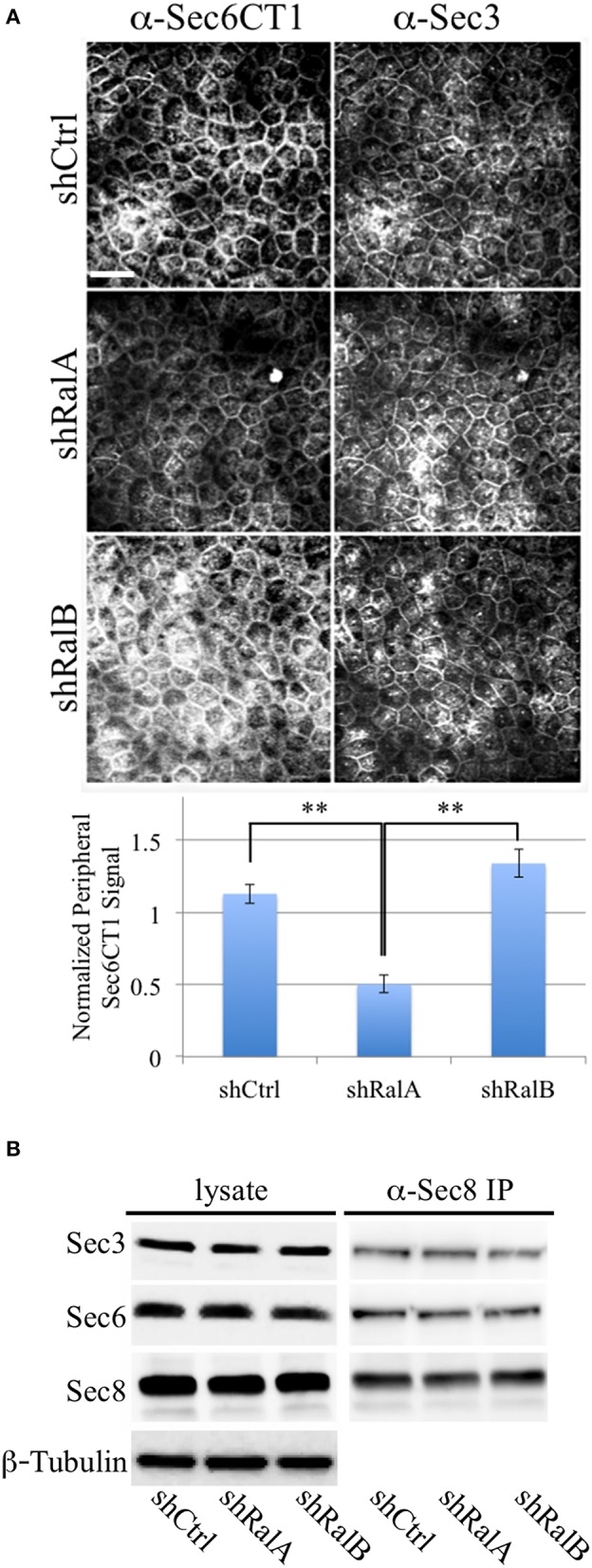
**RalA regulates plasma membrane exocyst configuration**. **(A)** Accessibility of Sec6 CT1 epitopes is reduced when RalA, but not RalB, expression is suppressed. *(Top)* MDCK cells stably expressing a non-specific short hairpin RNA (shCtrl), or a hairpin specific for either RalA (shRalA) or RalB (shRalB), were fixed and labeled with a cocktail of mouse monoclonal antibodies to Sec6 domain CT1 (mAbs 4C8, 9E9, 9H5, and 15C2). Cells were co-labeled with rabbit polyclonal antisera against Sec3. Bound antibodies were revealed by indirect immunofluorescence, following labeling with FITC-anti-mouse and Texas Red-anti-rabbit antibodies. *(Bottom)* Antibody labeling intensities at the plasma membrane were quantified as described in Material and Methods. Note that Sec6CT labeling, which is primarily concentrated at the lateral plasma membrane of control cells, is diminished upon RalA, but not RalB reduction. This likely reflects differences in Sec6 conformation, rather than exocyst localization, because plasma membrane Sec3 labeling is not changed following either knockdown. Bar = 20 micron, ^**^p < 0.05. **(B)** Levels of Sec3/6/8 complex are not altered following suppression of either RalA or RalB expression. RIPA extracts of MDCK shCtrl, shRalA, or shRalB cells were immunoprecipitated with anti-Sec8-bound protein A sepharose. Equal fractions of input lysates and immunoprecipitates were resolved by SDS-PAGE and immunoblotted with rabbit anti-Sec3, mouse anti-Sec8(8F12) or mouse anti-Sec6(10D11) antibodies. An immunoblot probed for β-tubulin is shown as a loading control.

We hypothesize that Ral GTPases regulate exocyst activities by facilitating association and/or dissociation of additional proteins with the holocomplex scaffold. Ral-mediated assembly or disassembly of exocyst super-complexes is expected to involve structural changes that render Sec6 epitopes more or less accessible to specific mAbs. To test this prediction, we compared binding of antibodies to the Sec6 CT1 subdomain in cells in which expression of either RalA or RalB had been suppressed. This analysis revealed that exposure of Sec6 CT1 epitopes at the plasma membrane was significantly decreased when expression of RalA, but not RalB, was reduced (Figure 9A). Loss of peripheral Sec6 CT1 labeling in cells lacking RalA was not due to inefficient recruitment of exocyst complexes to the plasma membrane, because peripheral Sec3 labeling was similar in control, shRalA and shRalB cells (Figure 9A). Furthermore, expression levels of Sec3, Sec6 and Sec8, and their assembly into exocyst complexes was unaffected by suppressing either RalA or RalB expression (Figure 9B). Collectively, these results support the conclusion that exocyst *conformation*, rather than expression, assembly or recruitment of the complex to the plasma membrane is regulated by RalA activity.

### Epitope accessibility is correlated with stability of Sec6 interactions with other exocyst subunits

**Figure 10:**
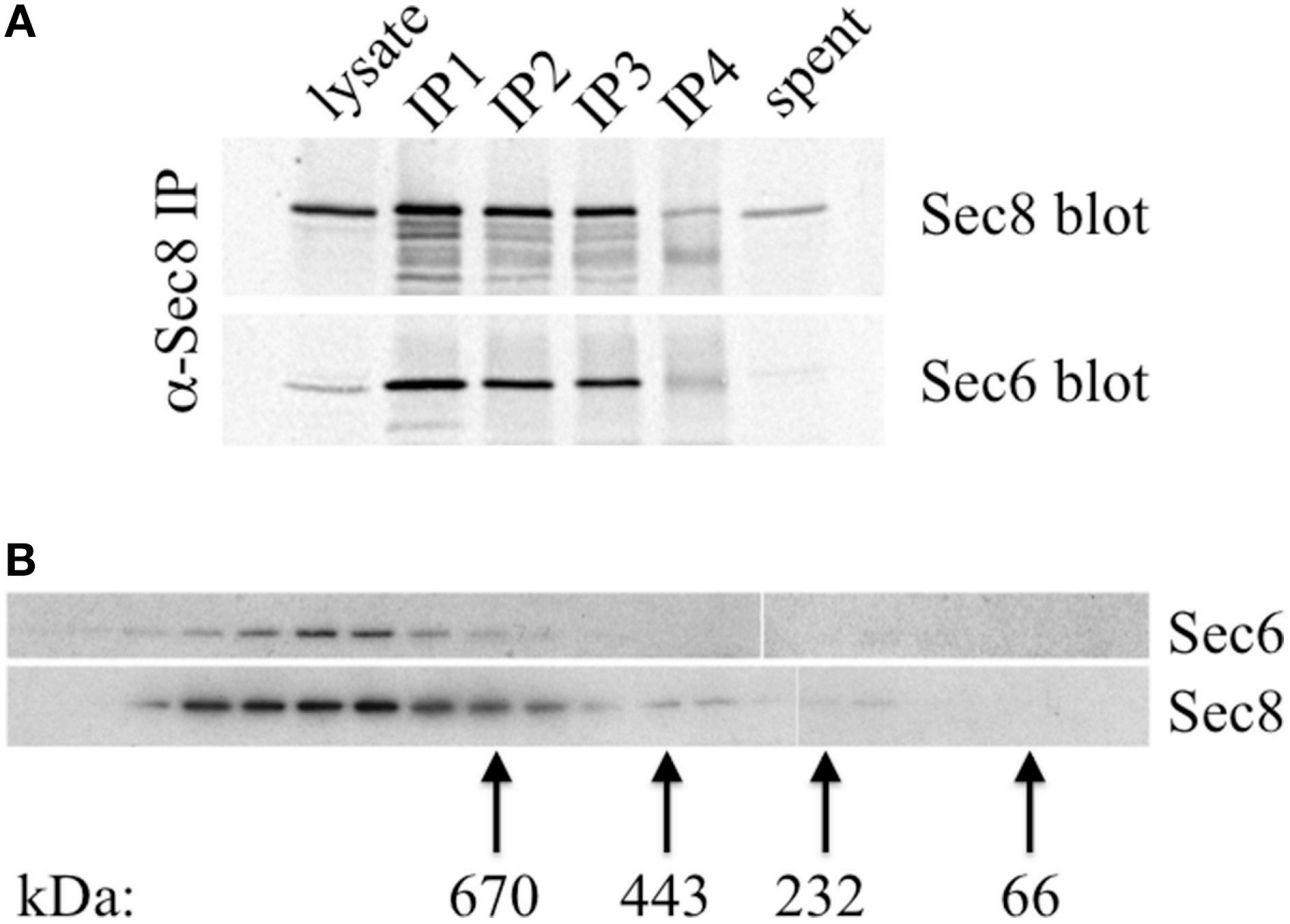
**Sec6 is quantitatively associated with Sec8 in high molecular weight complexes in MDCK cells**. **(A)** All endogenous Sec6 is bound to Sec8. MDCK RIPA extracts were immunoprecipitated 4 times with anti-Sec8-bound protein A sepharose. Input lysate represents 10% of starting material. Depleted lysate (“spent”) represents 50% of the final post-immunoprecipitation supernatant. Samples were resolved by SDS-PAGE and immunoblotted with anti-Sec8(8F12) and anti-Sec6(10D11) antibodies. **(B)** Detergent extracts of polarized MDCK cells were fractionated by Superose 6 FPLC as described in Experimental Procedures. Fractions 9–27 were divided into equal aliquots, separated by SDS-PAGE, and transferred to Immobilon P membranes. Membranes were probed with anti-Sec8(8F12) and anti-Sec6(10D11) antibodies. Elution peaks of globular protein standards with known molecular weights were also determined: thyroglobulin, Mr = 669,000 (fraction 16); apoferritin, Mr = 443,000 (fraction 19); catalase, Mr = 232,000 (fraction 22); bovine serum albumin, Mr = 66,000 (fraction 25).

A possible explanation for why different Sec6 epitopes are either exposed or concealed in cells is that Sec6 exists in different complexes, and that spatially regulated associations with other proteins either masks or reveals epitopes in different parts of the cell or at different stages of polarity development. Importantly, epithelial cells do not accumulate a significant pool of free, monomeric Sec6. Quantitative immunoprecipiation of Sec8 recovers nearly all of the Sec6 in cell extracts (Figure 10A). This is consistent with a recent report that Sec6 and Sec8 form a stable subunit pair in budding yeast (Heider et al., 2016). Gel filtration chromatography of MDCK cell lysates shows that this Sec6/8 complex is always associated with high molecular weight assemblies (Figure 10B). Therefore, the diversity of immunolabeling patterns observed likely reflects heterogeneity within multiple populations of large protein assemblies containing Sec6.

**Figure 11:**
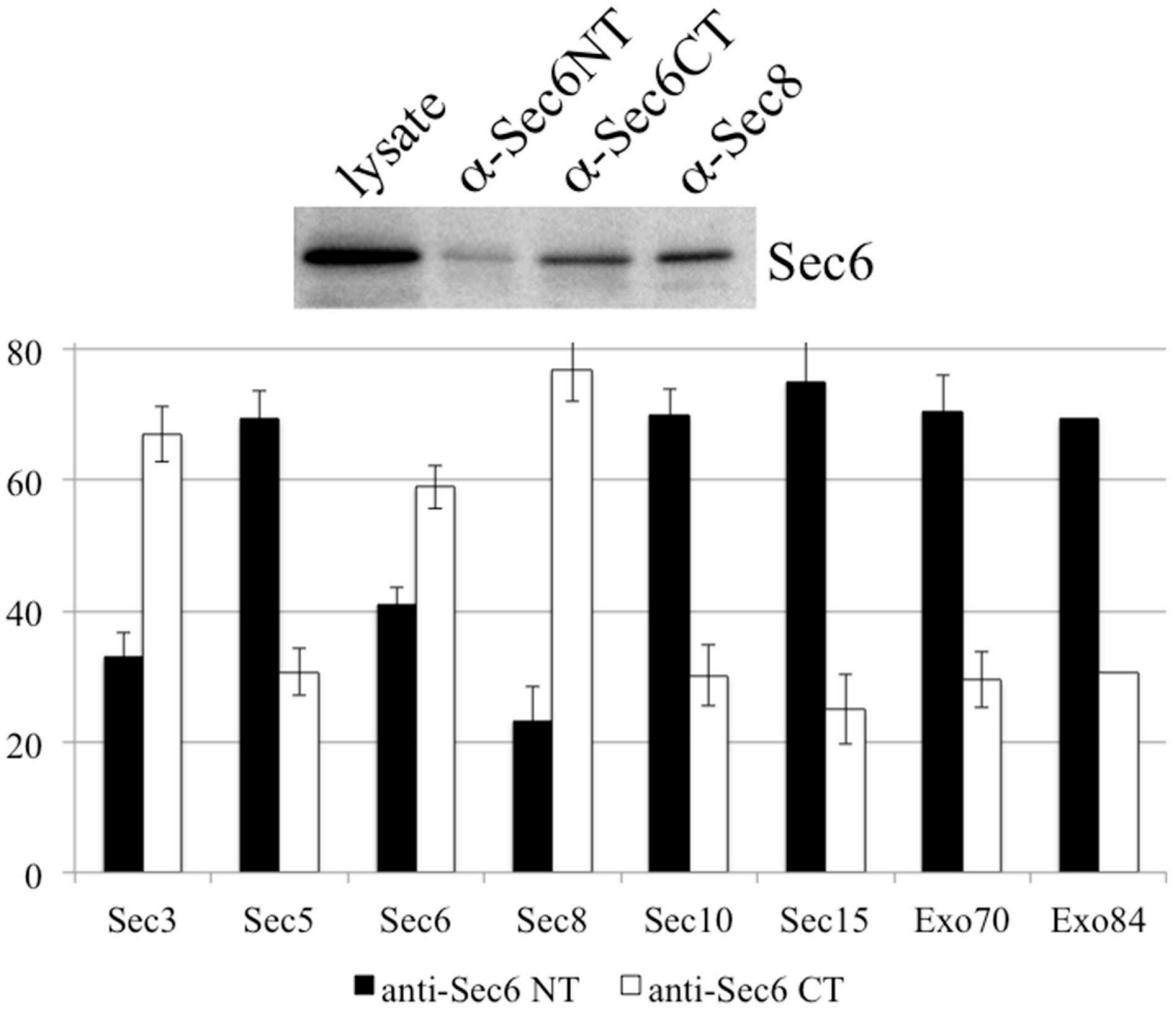
**Antibodies to different Sec6 domains differentially co-precipitate other exocyst subunits**. Cocktails of antibodies specific for epitopes in either the Sec6 N-terminus (8F9, 10D11, 13F10, 8A11, 1F5, 7C6, 16G4, and 22F) or C-terminus (9E9, 9H5, 10C3, 2B12, 3F3, 8E6, 11A2, and 8A5) were used to immunoprecipitate exocyst complexes from MDCK lysates. Precipitates were resolved by SDS-PAGE and immunoblotted with antibodies to either Sec6 or indicated exocyst subunits. Amounts of each subunit that were recovered were normalized to amounts of Sec6 recovered in each immunoprecipitate, and are expressed as a fraction of the total immunoprecipitated by both Sec6 NT and CT antibody cocktails. Bars represent means of three independent experiments, and error bars represent standard deviations.

It is possible that Sec6 epitopes are differentially accessible because exocyst holocomplexes undergo context-specific conformational changes or shifts in inter-molecular interactions that impact Sec6, such that individual subdomains are either exposed or concealed. To test whether compartment-specific exposure of different epitopes was correlated with differential stability of interactions between Sec6 and other subunits, Sec6 was immunoprecipitated with cocktails of mAbs that bind epitopes in either the N-terminal or C-terminal halves of the protein, and the relative amounts of other exocyst subunits that co-precipitated was measured (Figure 11). mAbs to Sec6NT co-precipitated more Sec5, Sec10, Sec15, Exo70, and Exo84 than did those to Sec6CT. mAbs to Sec6CT, by contrast, co-precipitated more Sec3 and Sec8 than did those to Sec6NT. These results suggest that exocyst complexes may undergo structural transitions that conceal Sec6NT but expose Sec6CT when complexes are membrane-bound, and expose Sec6NT but conceal Sec6CT when complexes are cytosolic or nuclear.

## Discussion

Previous studies of exocyst localization in mammalian epithelial cells have revealed complexes at AJC (Grindstaff et al., 1998; Yeaman et al., 2004) and desmosomes (Andersen and Yeaman, 2010) on basolateral plasma membranes, developing apical plasma membranes (Bryant et al., 2010), *trans*-Golgi network (Yeaman et al., 2001), recycling endosomes (Folsch et al., 2003; Prigent et al., 2003; Oztan et al., 2007), primary cilia (Rogers et al., 2004), and centrosomes (Rogers et al., 2004; Andersen and Yeaman, 2010). Discrepancies in reported localizations may reflect differences in fixation and permeabilization procedures, specific antibodies used in the study, extent of cellular polarization, growth conditions of cells, existence of exocyst sub-complexes associated with different organelles and the possibility that exocyst complexes may exist in different conformational states. In this study, we have focused on a single exocyst subunit (Sec6), carefully mapped epitopes bound by more than 20 distinct mAbs and used them to probe subdomain accessibility in exocyst complexes associated with different cellular compartments.

A major finding of this work is that epitopes mapping to different segments of Sec6 are exposed or concealed in a spatially controlled fashion in epithelial cells. Sec6 is an obligate binding partner of Sec8, and is entirely associated with large multi-protein assemblies in MDCK cells. Nevertheless, anti-Sec6 mAbs detect epitopes on different parts of this protein at different subcellular localizations. Of equal importance, no single epitope is exposed at all of these sites. This suggests that the majority of Sec6 epitopes are concealed at most sites, and that only a small subset of epitopes are exposed and this occurs in a site-specific manner. Therefore, Sec6 epitopes and the mAbs that bind them define exocyst complexes associated with one, or a small subset of cellular compartments, where they are presumably engaged in only one activity.

We also note that discrete Sec6 epitopes may appear, disappear or redistribute as cells grow and differentiate. For example, antibodies to the NT1 domain label centrosomes, but only when cells are undergoing mitosis. In contrast, antibodies to the NT2 domain label nucleoli of interphase cells, but do not detect Sec6 anywhere in mitotic cells. Epitopes in the C-terminal domain appear to redistribute during epithelial cell polarity development. The CT2b segment is accessible for mAb 11A2 binding to a pool of Sec6 associated with vimentin filaments in non-polarized cells, and no plasma membrane labeling with this antibody is observed under these conditions. However, the same antibody reveals Sec6 associated with apical and basolateral domains of fully polarized cells. Similarly, the CT3 segment is accessible for mAb 8A5 binding to a pool of Sec6 associated with desmosomes in non-polarized cells and 2-D polarized cells on Transwells (Andersen and Yeaman, 2010). However, this antibody labels Sec6 in a continuous and uniform distribution along the basolateral plasma membrane of fully polarized cells grown in Matrigel. Antibodies to the CT1 domain detect Sec6 at newly forming adhesive junctions and mark a complex that becomes restricted to the AJC in polarized cells grown on Transwell filters (Grindstaff et al., 1998; Yeaman et al., 2004), but do not specifically label Sec6 in fully polarized 3-D cysts. We acknowledge that care must be taken to distinguish between physical redistribution of exocyst complexes and changes in conformation and/or molecular associations that reveal or conceal epitopes at a given site, because both are likely to accompany epithelial polarity development. Because gentle pre-extraction with non-ionic detergent exposes Sec6 CT1 epitopes at the AJC of MDCK cysts, we conclude that either intramolecular interactions within the exocyst holocomplex or associations with accessory proteins mask these epitopes in fully polarized cells. Such molecular rearrangements are likely to be regulated, at least in part, by RalA, because suppressing expression of this GTPase significantly diminished binding of CT1-specific mAbs to plasma membranes without affecting the expression, assembly or recruitment of exocyst complexes to this site.

We anticipate that a thorough analysis of epitope accessibility of each subunit will reveal important details about exocyst structure, function and regulation during its involvement in many different cellular processes. A complete understanding of these functions will require comprehensive identification of accessory proteins with which exocyst holocomplexes interact at different sites. We have shown that Sec6 epitopes, and the mAbs that bind them, define exocyst complexes associated with one (or a small subset of) cellular compartments. As such, the library of Sec6 mAbs is likely to be an important resource for the study of different cohorts of exocyst complexes engaged in different cellular processes.

## Author contributions

SI and CY made substantial contributions to conception, design, acquisition, analysis, and interpretation of data for the work. SI, SH, and CY drafted the work and revised it critically for intellectual content. SI, SH, and CY approved the final version to be published and agreed to be accountable for all aspects of the work.

## Funding

This work was supported by a grant from the National Institutes of Health (GM067002) to CY.

### Conflict of interest statement

The authors declare that the research was conducted in the absence of any commercial or financial relationships that could be construed as a potential conflict of interest.

## References

[B1] Andersen N. J. Yeaman C. (2010). Sec3-containing exocyst complex is required for desmosome assembly in mammalian epithelial cells. Mol. Biol. Cell 21, 152–164. 10.1091/mbc.E09-06-0459 19889837 PMC2801709

[B2] Beronja S. Laprise P. Papoulas O. Pellikka M. Sisson J. Tepass U. (2005). Essential function of Drosophila Sec6 in apical exocytosis of epithelial photoreceptor cells. J. Cell Biol. 169, 635–646. 10.1083/jcb.200410081 15897260 PMC2171699

[B3] Bodemann B. O. Orvedahl A. Cheng T. Ram R. R. Ou Y. H. Formstecher E.. (2011). RalB and the exocyst mediate the cellular starvation response by direct activation of autophagosome assembly. Cell 144, 253–267. 10.1016/j.cell.2010.12.018 21241894 PMC3038590

[B4] Bryant D. M. Datta A. Rodriguez-Fraticelli A. E. Peranen J. Martin-Belmonte F. Mostov K. E. (2010). A molecular network for de novo generation of the apical surface and lumen. Nat. Cell Biol. 12, 1035–1045. 10.1038/ncb2106 20890297 PMC2975675

[B5] Cascone I. Selimoglu R. Ozdemir C. Del Nery E. Yeaman C. White M.. (2008). Distinct roles of RalA and RalB in the progression of cytokinesis are supported by distinct RalGEFs. EMBO J. 27, 2375–2387. 10.1038/emboj.2008.166 18756269 PMC2543054

[B6] Chen X. W. Leto D. Xiao J. Goss J. Wang Q. Shavit J. A.. (2011). Exocyst function is regulated by effector phosphorylation. Nat. Cell Biol. 13, 580–588. 10.1038/ncb2226 21516108 PMC3904505

[B7] Chien Y. Kim S. Bumeister R. Loo Y. M. Kwon S. W. Johnson C. L.. (2006). RalB GTPase-mediated activation of the IkappaB family kinase TBK1 couples innate immune signaling to tumor cell survival. Cell 127, 157–170. 10.1016/j.cell.2006.08.034 17018283

[B8] Das A. Gajendra S. Falenta K. Oudin M. J. Peschard P. Feng S.. (2014). RalA promotes a direct exocyst-Par6 interaction to regulate polarity in neuronal development. J. Cell Sci. 127, 686–699. 10.1242/jcs.145037 24284074 PMC4007768

[B9] Das A. Guo W. (2011). Rabs and the exocyst in ciliogenesis, tubulogenesis and beyond. Trends Cell Biol. 21, 383–386. 10.1016/j.tcb.2011.03.006 21550243 PMC3128673

[B10] Dubuke M. L. Maniatis S. Shaffer S. A. Munson M. (2015). The Exocyst Subunit Sec6 Interacts with Assembled Exocytic SNARE Complexes. J. Biol. Chem. 290, 28245–28256. 10.1074/jbc.M115.673806 26446795 PMC4653681

[B11] Fielding A. B. Schonteich E. Matheson J. Wilson G. Yu X. Hickson G. R.. (2005). Rab11-FIP3 and FIP4 interact with Arf6 and the exocyst to control membrane traffic in cytokinesis. EMBO J. 24, 3389–3399. 10.1038/sj.emboj.7600803 16148947 PMC1276165

[B12] Folsch H. Pypaert M. Maday S. Pelletier L. Mellman I. (2003). The AP-1A and AP-1B clathrin adaptor complexes define biochemically and functionally distinct membrane domains. J. Cell Biol. 163, 351–362. 10.1083/jcb.200309020 14581457 PMC2173537

[B13] France Y. E. Boyd C. Coleman J. Novick P. J. (2006). The polarity-establishment component Bem1p interacts with the exocyst complex through the Sec15p subunit. J. Cell Sci. 119, 876–888. 10.1242/jcs.02849 16478783

[B14] Goehring A. S. Pedroja B. S. Hinke S. A. Langeberg L. K. Scott J. D. (2007). MyRIP anchors protein kinase A to the exocyst complex. J. Biol. Chem. 282, 33155–33167. 10.1074/jbc.M705167200 17827149 PMC3508720

[B15] Grindstaff K. K. Yeaman C. Anandasabapathy N. Hsu S. C. Rodriguez-Boulan E. Scheller R. H.. (1998). Sec6/8 complex is recruited to cell-cell contacts and specifies transport vesicle delivery to the basal-lateral membrane in epithelial cells. Cell 93, 731–740. 10.1016/S0092-8674(00)81435-X 9630218

[B16] Gromley A. Yeaman C. Rosa J. Redick S. Chen C. T. Mirabelle S.. (2005). Centriolin anchoring of exocyst and SNARE complexes at the midbody is required for secretory-vesicle-mediated abscission. Cell 123, 75–87. 10.1016/j.cell.2005.07.027 16213214

[B17] Hase K. Kimura S. Takatsu H. Ohmae M. Kawano S. Kitamura H.. (2009). M-Sec promotes membrane nanotube formation by interacting with Ral and the exocyst complex. Nat. Cell Biol. 11, 1427–1432. 10.1038/ncb1990 19935652

[B18] Hazelett C. C. Sheff D. Yeaman C. (2011). RalA and RalB differentially regulate development of epithelial tight junctions. Mol. Biol. Cell 22, 4787–4800. 10.1091/mbc.E11-07-0657 22013078 PMC3237622

[B19] Heider M. R. Gu M. Duffy C. M. Mirza A. M. Marcotte L. L. Walls A. C.. (2016). Subunit connectivity, assembly determinants and architecture of the yeast exocyst complex. Nat. Struct. Mol. Biol. 23, 59–66. 10.1038/nsmb.3146 26656853 PMC4752824

[B20] Hsu S. C. Hazuka C. D. Roth R. Foletti D. L. Heuser J. Scheller R. H. (1998). Subunit composition, protein interactions, and structures of the mammalian brain sec6/8 complex and septin filaments. Neuron 20, 1111–1122. 10.1016/S0896-6273(00)80493-6 9655500

[B21] Hsu S. C. Ting A. E. Hazuka C. D. Davanger S. Kenny J. W. Kee Y.. (1996). The mammalian brain rsec6/8 complex. Neuron 17, 1209–1219. 10.1016/S0896-6273(00)80251-2 8982167

[B22] Ishikawa H. Ma Z. Barber G. N. (2009). STING regulates intracellular DNA-mediated, type I interferon-dependent innate immunity. Nature 461, 788–792. 10.1038/nature08476 19776740 PMC4664154

[B23] Jafar-Nejad H. Andrews H. K. Acar M. Bayat V. Wirtz-Peitz F. Mehta S. Q.. (2005). Sec15, a component of the exocyst, promotes notch signaling during the asymmetric division of Drosophila sensory organ precursors. Dev. Cell 9, 351–363. 10.1016/j.devcel.2005.06.010 16137928

[B24] Jin Y. Sultana A. Gandhi P. Franklin E. Hamamoto S. Khan A. R.. (2011). Myosin V transports secretory vesicles via a Rab GTPase cascade and interaction with the exocyst complex. Dev. Cell 21, 1156–1170. 10.1016/j.devcel.2011.10.009 22172676 PMC3241923

[B25] Kee Y. Yoo J. S. Hazuka C. D. Peterson K. E. Hsu S. C. Scheller R. H. (1997). Subunit structure of the mammalian exocyst complex. Proc. Natl. Acad. Sci. U.S.A. 94, 14438–14443. 10.1073/pnas.94.26.14438 9405631 PMC25013

[B26] Lalli G. (2009). RalA and the exocyst complex influence neuronal polarity through PAR-3 and aPKC. J. Cell Sci. 122, 1499–1506. 10.1242/jcs.044339 19383721

[B27] Lipschutz J. H. Guo W. O'Brien L. E. Nguyen Y. H. Novick P. Mostov K. E. (2000). Exocyst is involved in cystogenesis and tubulogenesis and acts by modulating synthesis and delivery of basolateral plasma membrane and secretory proteins. Mol. Biol. Cell 11, 4259–4275. 10.1091/mbc.11.12.4259 11102522 PMC15071

[B28] Liu J. Yue P. Artym V. V. Mueller S. C. Guo W. (2009). The role of the exocyst in matrix metalloproteinase secretion and actin dynamics during tumor cell invadopodia formation. Mol. Biol. Cell 20, 3763–3771. 10.1091/mbc.E08-09-0967 19535457 PMC2777935

[B29] Luo G. Zhang J. Guo W. (2014). The role of Sec3p in secretory vesicle targeting and exocyst complex assembly. Mol. Biol. Cell 25, 3813–3822. 10.1091/mbc.E14-04-0907 25232005 PMC4230786

[B30] Medkova M. France Y. E. Coleman J. Novick P. (2006). The rab exchange factor Sec2p reversibly associates with the exocyst. Mol. Biol. Cell 17, 2757–2769. 10.1091/mbc.E05-10-0917 16611746 PMC1474791

[B31] Mehta S. Q. Hiesinger P. R. Beronja S. Zhai R. G. Schulze K. L. Verstreken P.. (2005). Mutations in Drosophila sec15 reveal a function in neuronal targeting for a subset of exocyst components. Neuron 46, 219–232. 10.1016/j.neuron.2005.02.029 15848801

[B32] Mohammadi S. Isberg R. R. (2013). Cdc42 interacts with the exocyst complex to promote phagocytosis. J. Cell Biol. 200, 81–93. 10.1083/jcb.201204090 23295348 PMC3542798

[B33] Morgera F. Sallah M. R. Dubuke M. L. Gandhi P. Brewer D. N. Carr C. M.. (2012). Regulation of exocytosis by the exocyst subunit Sec6 and the SM protein Sec1. Mol. Biol. Cell 23, 337–346. 10.1091/mbc.E11-08-0670 22114349 PMC3258177

[B34] Munson M. Novick P. (2006). The exocyst defrocked, a framework of rods revealed. Nat. Struct. Mol. Biol. 13, 577–581. 10.1038/nsmb1097 16826234

[B35] Nichols C. D. Casanova J. E. (2010). Salmonella-directed recruitment of new membrane to invasion foci via the host exocyst complex. Curr. Biol. 20, 1316–1320. 10.1016/j.cub.2010.05.065 20579884 PMC2910195

[B36] Overgaard C. E. Sanzone K. M. Spiczka K. S. Sheff D. R. Sandra A. Yeaman C. (2009). Deciliation is associated with dramatic remodeling of epithelial cell junctions and surface domains. Mol. Biol. Cell 20, 102–113. 10.1091/mbc.E08-07-0741 19005211 PMC2613083

[B37] Oztan A. Silvis M. Weisz O. A. Bradbury N. A. Hsu S. C. Goldenring J. R.. (2007). Exocyst requirement for endocytic traffic directed toward the apical and basolateral poles of polarized MDCK cells. Mol. Biol. Cell 18, 3978–3992. 10.1091/mbc.E07-02-0097 17686995 PMC1995710

[B38] Parrini M. C. Sadou-Dubourgnoux A. Aoki K. Kunida K. Biondini M. Hatzoglou A.. (2011). SH3BP1, an exocyst-associated RhoGAP, inactivates Rac1 at the front to drive cell motility. Mol. Cell 42, 650–661. 10.1016/j.molcel.2011.03.032 21658605 PMC3488376

[B39] Pathak R. Delorme-Walker V. D. Howell M. C. Anselmo A. N. White M. A. Bokoch G. M.. (2012). The microtubule-associated Rho activating factor GEF-H1 interacts with exocyst complex to regulate vesicle traffic. Dev. Cell 23, 397–411. 10.1016/j.devcel.2012.06.014 22898781 PMC3422510

[B40] Prigent M. Dubois T. Raposo G. Derrien V. Tenza D. Rosse C.. (2003). ARF6 controls post-endocytic recycling through its downstream exocyst complex effector. J. Cell Biol. 163, 1111–1121. 10.1083/jcb.200305029 14662749 PMC2173613

[B41] Reaves B. Banting G. (1992). Perturbation of the morphology of the trans-Golgi network following Brefeldin A treatment: redistribution of a TGN-specific integral membrane protein, TGN38. J. Cell Biol. 116, 85–94. 10.1083/jcb.116.1.85 1730751 PMC2289270

[B42] Rittmeyer E. N. Daniel S. Hsu S. C. Osman M. A. (2008). A dual role for IQGAP1 in regulating exocytosis. J. Cell Sci. 121, 391–403. 10.1242/jcs.016881 18216334

[B43] Rogers K. K. Wilson P. D. Snyder R. W. Zhang X. Guo W. Burrow C. R.. (2004). The exocyst localizes to the primary cilium in MDCK cells. Biochem. Biophys. Res. Commun. 319, 138–143. 10.1016/j.bbrc.2004.04.165 15158452

[B44] Rosse C. Hatzoglou A. Parrini M. C. White M. A. Chavrier P. Camonis J. (2006). RalB mobilizes the exocyst to drive cell migration. Mol. Cell. Biol. 26, 727–734. 10.1128/MCB.26.2.727-734.2006 16382162 PMC1346891

[B45] Sakurai-Yageta M. Recchi C. Le Dez G. Sibarita J. B. Daviet L. Camonis J.. (2008). The interaction of IQGAP1 with the exocyst complex is required for tumor cell invasion downstream of Cdc42 and RhoA. J. Cell Biol. 181, 985–998. 10.1083/jcb.200709076 18541705 PMC2426946

[B46] Simicek M. Lievens S. Laga M. Guzenko D. Aushev V. N. Kalev P.. (2013). The deubiquitylase USP33 discriminates between RALB functions in autophagy and innate immune response. Nat. Cell Biol. 15, 1220–1230. 10.1038/ncb2847 24056301

[B47] Sivaram M. V. Saporita J. A. Furgason M. L. Boettcher A. J. Munson M. (2005). Dimerization of the exocyst protein Sec6p and its interaction with the t-SNARE Sec9p. Biochemistry 44, 6302–6311. 10.1021/bi048008z 15835919

[B48] Songer J. A. Munson M. (2009). Sec6p anchors the assembled exocyst complex at sites of secretion. Mol. Biol. Cell 20, 973–982. 10.1091/mbc.E08-09-0968 19073882 PMC2633393

[B49] Spiczka K. S. Yeaman C. (2008). Ral-regulated interaction between Sec5 and paxillin targets Exocyst to focal complexes during cell migration. J. Cell Sci. 121, 2880–2891. 10.1242/jcs.031641 18697830 PMC4445373

[B50] Stalder D. Novick P. J. (2016). The casein kinases Yck1p and Yck2p act in the secretory pathway, in part, by regulating the Rab exchange factor Sec2p. Mol. Biol. Cell 27, 686–701. 10.1091/mbc.E15-09-0651 26700316 PMC4750927

[B51] Stevenson B. R. Begg D. A. (1994). Concentration-dependent effects of cytochalasin D on tight junctions and actin filaments in MDCK epithelial cells. J. Cell Sci. 107 (Pt 3), 367–375. 8006058 10.1242/jcs.107.3.367

[B52] Stewart D. B. Nelson W. J. (1997). Identification of four distinct pools of catenins in mammalian cells and transformation-dependent changes in catenin distributions among these pools. J. Biol. Chem. 272, 29652–29662. 10.1074/jbc.272.47.29652 9368032

[B53] Torres M. J. Pandita R. K. Kulak O. Kumar R. Formstecher E. Horikoshi N.. (2015). Role of the Exocyst Complex Component Sec6/8 in Genomic Stability. Mol. Cell. Biol. 35, 3633–3645. 10.1128/MCB.00768-15 26283729 PMC4589602

[B54] Vega I. E. Hsu S. C. (2001). The exocyst complex associates with microtubules to mediate vesicle targeting and neurite outgrowth. J. Neurosci. 21, 3839–3848. 11356872 10.1523/JNEUROSCI.21-11-03839.2001 PMC3674029

[B55] Vik-Mo E. O. Oltedal L. Hoivik E. A. Kleivdal H. Eidet J. Davanger S. (2003). Sec6 is localized to the plasma membrane of mature synaptic terminals and is transported with secretogranin II-containing vesicles. Neuroscience 119, 73–85. 10.1016/S0306-4522(03)00065-4 12763070

[B56] Wang S. Hsu S. C. (2003). Immunological characterization of exocyst complex subunits in cell differentiation. Hybrid. Hybridomics 22, 159–164. 10.1089/153685903322286575 12954101

[B57] Yeaman C. (2003). Ultracentrifugation-based approaches to study regulation of Sec6/8 (exocyst) complex function during development of epithelial cell polarity. Methods 30, 198–206. 10.1016/S1046-2023(03)00026-4 12798134

[B58] Yeaman C. Grindstaff K. K. Nelson W. J. (2004). Mechanism of recruiting Sec6/8 (exocyst) complex to the apical junctional complex during polarization of epithelial cells. J. Cell Sci. 117, 559–570. 10.1242/jcs.00893 14709721 PMC3368615

[B59] Yeaman C. Grindstaff K. K. Wright J. R. Nelson W. J. (2001). Sec6/8 complexes on trans-Golgi network and plasma membrane regulate late stages of exocytosis in mammalian cells. J. Cell Biol. 155, 593–604. 10.1083/jcb.200107088 11696560 PMC2198873

[B60] Zuo X. Guo W. Lipschutz J. H. (2009). The exocyst protein Sec10 is necessary for primary ciliogenesis and cystogenesis *in vitro*. Mol. Biol. Cell 20, 2522–2529. 10.1091/mbc.E08-07-0772 19297529 PMC2682593

[B61] Zuo X. Zhang J. Zhang Y. Hsu S. C. Zhou D. Guo W. (2006). Exo70 interacts with the Arp2/3 complex and regulates cell migration. Nat. Cell Biol. 8, 1383–1388. 10.1038/ncb1505 17086175

